# Ki-67 is a strong prognostic marker of non-small cell lung cancer when tissue heterogeneity is considered

**DOI:** 10.1186/1472-6890-14-23

**Published:** 2014-05-13

**Authors:** Kazuhiro Tabata, Tomonori Tanaka, Tomayoshi Hayashi, Takashi Hori, Sayuri Nunomura, Suguru Yonezawa, Junya Fukuoka

**Affiliations:** 1 Department of Pathology, Nagasaki University Graduate School of Biomedical Sciences, Nagasaki, Japan; 2 Department of Pathology, Nagasaki University Hospital, Nagasaki, Japan; 3 Department of Surgical Pathology, Laboratory of Pathology, Toyama, Japan; 4 Department of Pathology, Toyama University Hospital, 2630 Sugitani, Toyama 930-0194, Japan; 5 Department of Human Pathology, Field of Oncology, Kagoshima University Graduate School of Medical and Dental Sciences, Kagoshima, Japan

**Keywords:** Tissue microarray, Tissue heterogeneity, Expression, Biomarkers, Pathology, Lung cancer

## Abstract

**Background:**

Ki-67 expression is a well-established prognostic marker in various cancers. However, Ki-67 expression is also known as being heterogeneous. We investigated the prognostic significance of Ki-67 from the view of staining heterogeneity by the technique of Spiral Array.

**Methods:**

100 cases of resected lung cancer from Toyama university hospital archive were collected. Spiral Array blocks were generated out of 100 cases using 100 μm thick paraffin sections. Four μm thick sections of the Array block were stained for Ki-67. Staining results in each reel were scored for areas with lowest (LS), highest (HS), and average (AS) expression, exclusively in the cancer cells. Heterogeneity score (HeS) was designed as the difference between HS and LS. The scores were divided into four grades (0–3). Clinical information was collected, and the prognostic significance of Ki-67 was analyzed.

**Results:**

Pathological stage was available for 91 patients (43 stage IA, 22 stage IB, 2 stage IIA, 9 stage IIB, 13 stage IIIA, 1 stage IIIB, and 1 stage IV). The HS of Ki-67 score in non-small cell lung cancer was 3 in 17 cases, 2 in 27 cases, 1 in 28 cases, 0 in 21 cases, and 4 reels were lost. 78 cases had clinical follow up. 74 cases had all the information available and were analyzed for correlation between Ki-67 expression and survival. Cases with score 2 and 3 of HS and HeS showed significant poorer prognosis (both *P* < 0.001), whereas LS or AS did not show significance. The results were identical when analyzing adenocarcinoma and squamous cell carcinoma, separately. Cox multivariate analysis of Ki-67 showed that HS was an independent risk factor affecting overall survival.

**Conclusions:**

Ki-67 is a strong prognostic marker for non-small cell lung cancer when the degree of highest staining frequency or heterogeneity is considered.

## Background

Lung cancer has the highest incidence and mortality of major cancers throughout the world [1]. Stage is still the most important prognostic factor and histology provides limited prognostic value. Since outcomes can be different even among patients with the same disease-stage, it is important to evaluate additional factors that may help to identify the patients with resectable tumors who are at high risk for recurrence and could consequently benefit from adjuvant therapy. The proliferative rate has been demonstrated to be a prognostic marker in some tumors, and Ki-67 is a marker of proliferation associated nuclear antigen expressed in replicating cells during all phases of the cell cycle (G1, S, G2 and M), but not expressed in quiescent (G0) cells [2]. The immunohistochemical (IHC) expression of Ki-67 has been used for the assessment of tumor proliferation, and high levels of Ki-67 antigen have been reported to be associated with a poor prognosis in many malignancies including those associated with carcinoma of the breast, prostate, bladder and lymph nodes [3-7]. However, its variable positivity within a given tumor limits might be in part responsible for some controversial findings in the literature [8,9]. Presence of tissue heterogeneity in each cancer type may be clinically important. Therefore, heterogeneity should be evaluated separately for each cancer type before extrapolating the results in clinical practice. Lung cancer is reported to be highly heterogeneous, and approximately 80 percent of adenocarcinoma shows a mixed subtype [10]. The present investigation aimed to evaluate the clinical significance of Ki-67 expression for predicting the prognosis in non-small cell lung cancer (NSCLC) especially in regards to tissue heterogeneity.

We have recently developed a novel technique, “Spiral array”, in which each tissue array core consists of a reeled layer of tissue cut as a horizontal section from the donor block and not punching as a vertical cylinder core [11]. Use of this Spiral Array technique gives us the advantage of eliminating sampling bias due to representation of different areas of the lesion by tumor heterogeneity.

### Ethics statement

This study was approved by the Institutional Review Board of the Toyama university hospital (No.19-12).

## Methods

### Case selection

One hundred case of resected primary lung cancer with the consent of patients and approval by internal review board were collected from Toyama university hospital archive based on the diagnosis and the quality of the available tissue on the paraffin block. The specimens were obtained through radical surgery, excisional biopsy or tumor debulking. The hematoxylin and eosin (H&E) slides from each case were reviewed and histologically classified according to the 2004 WHO histological classification of Lung Cancer by TT and JF [12]. Clinical information including follow-up status, Brinkman index, smoking history, and treatment of neoadjuvant or/and adjuvant therapy were also gathered. On collecting this cohort, we formed it anonymized.

### Construction of spiral arrays

**Figure 1:**
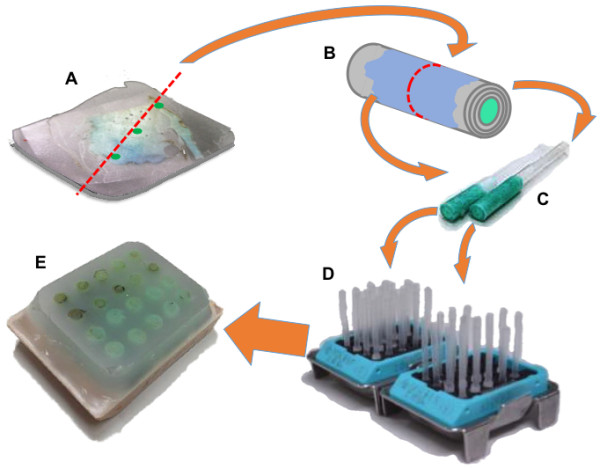
**Schematic figure indicating composition process of Spiral Array. A)** 100 μm-thick sections are cut from the donor blocks. **B)** The sections are then reeled on the plastic core. **C)** The subcylindrical cores were cut in the center of the reel. **D)** Each subcylindrical reel was vertically inserted into a tissue-holding-cassette. **E)** By pouring a melted paraffin, Spiral Array block is completed.

**Figure 2:**
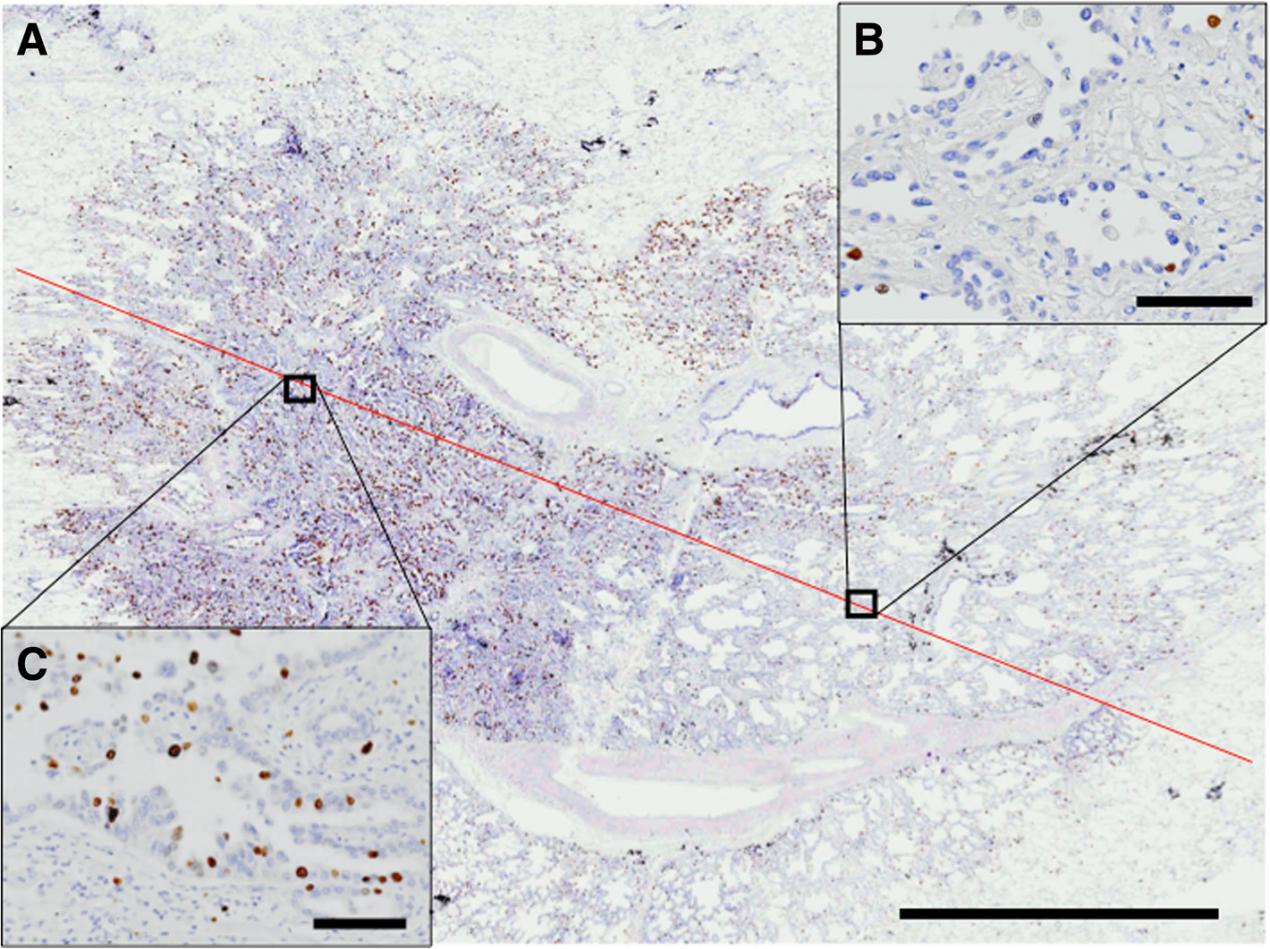
**Reflection of tissue heterogeneity by Spiral Array. A)** Ki-67 immunohistochemical staining of lung adenocarcinoma shows variable amounts of positive cells within the slide. Red line indicates the area of coverage inside the Spiral Array (bar = 5 mm). Both high expression area of Ki-67 **(B)** and low expression area of Ki-67 **(C)** are included inside the same reel of Spiral Array.

Formalin fixed paraffin embedded blocks from one hundred cases of lung cancer were collected from the pathology archives at Toyama University Hospital. Spiral Arrays were constructed as reported [Figure 1] [11,13]. Spiral Arrays covers morphological variations included in the one axis of the donor paraffin block, wider area than conventional tissue microarrays, and addresses the issue of tissue heterogeneity [Figure 2]. The blocks were heated to around 40°C on the surface, and 100 μm-thick sections were cut with a standard microtome. From the view of antigen preservation in the paraffin block, the surface layers of the original blocks were discarded. Then, the thick paraffin sections were applied to semi-auto Spiral Array Constructor (Sakura Finetek Japan, Tokyo). In the Array constructor, the sections were reeled on the plastic core tubes of 3 mm diameter followed by heating and cooling steps. The subcylinders were cut in the center of the reels. Each subcylindrical reel was vertically inserted into a tissue-holding-cassette made of Acrylonitrile Butadiene Styrene polymer with 20 holes, which are 3.3 mm diameter (Sakura Finetek Japan, Tokyo) [Figure 1]. The plastic cassettes were placed on the metal molds (Sakura Finetek Japan, Tokyo) where bottom surface of the molds were covered by double sided adhesive tapes. The edges of inserted subcylindrical reels were adhered by the tape. Melted paraffin (Paraffin wax II60, Sakura Finetek, Tokyo, Japan) at 65°C was poured into the cassette to re-embed the reels, and was followed by a cooling down period. Lastly, after removing the molds and adhesive tape, the Spiral Array block was sectioned at 4 μm.

### Immunohistochemical staining

Four μm thick sections of the Array blocks were stained with Ki-67. Immunohistochemical staining was performed using Ventana Benchmark XT (Ventana, Tucson, AZ) automated slide preparation system, and used a rabbit monoclonal antibody to Ki-67 (clone 30–9, Ventana, Tucson, AZ).

### Scoring of the staining results and statistical analysis

**Figure 3:**
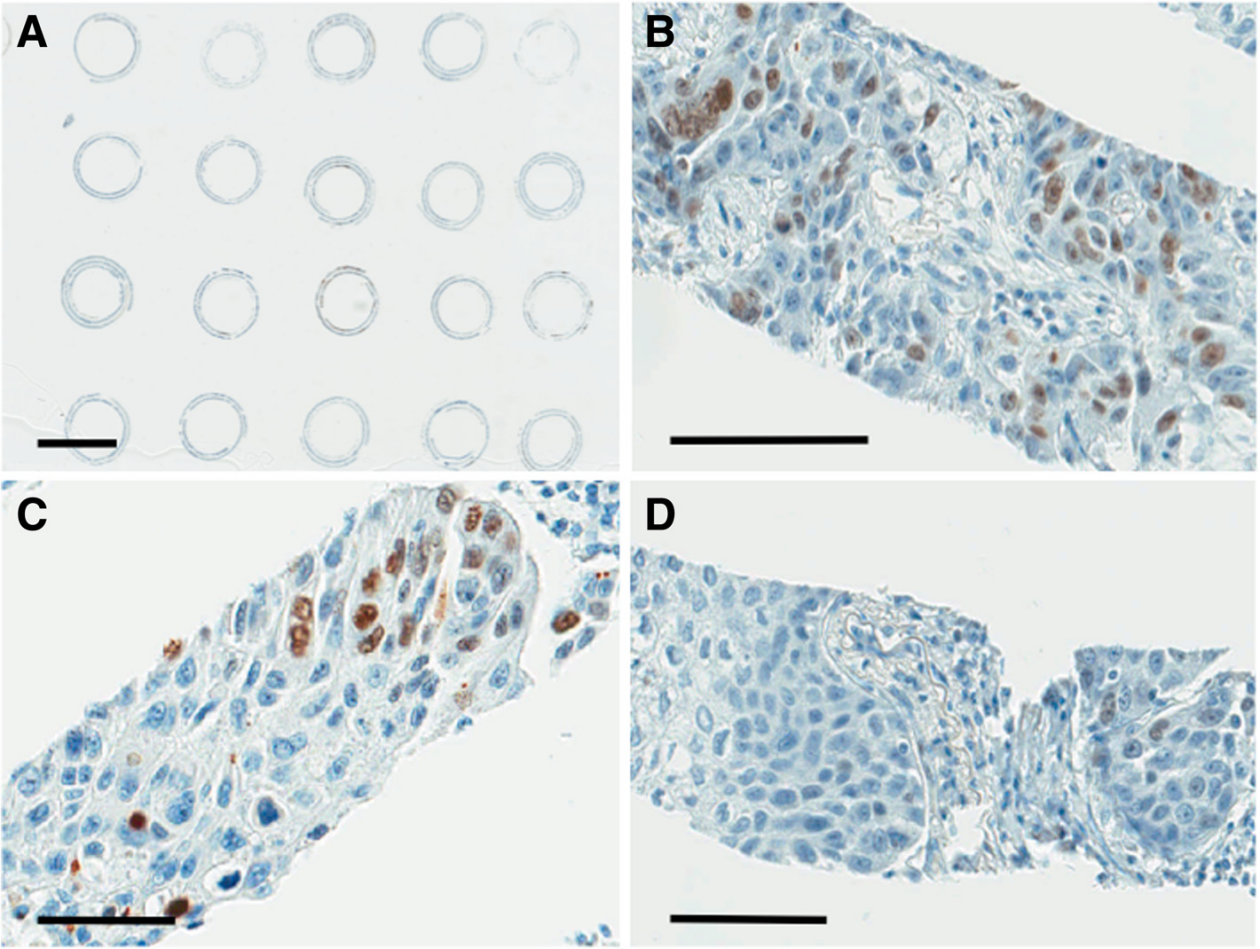
**Grading of Ki-67 immunohistochemical staining on Spiral Array.** Ki-67 positive ratio of tumor cells in a high power field (×400 magnification). Tumor cells with stained nucleus by the diaminobenzidine tetrahydrochloride, regardless their intensities, are count as positive. **A)** Scanning view of the Spiral Array slide stained with Ki-67. (bar = 5 mm). **B-D)** Examples of reels showing Ki-67 staining of Grade 3, > 30% **(B)**; Grade 2, 11-30% **(C)**; Grade 1, 1-10% **(D)**. (bars in B, C, D = 100 μm).

The reels were first observed at low magnification for determining the areas of highest and lowest expression of Ki-67, and designated as Highest Score (HS) and Lowest Score (LS), respectively. These areas were further analyzed at a single high power field (HPF, 400× magnification) and the staining scores were determined. Average of staining score in a whole reel was defined as Average Score (AS). Ki-67 expression was defined as the percent of Ki-67-positive tumor cells divided by the total number of tumor cells within one HPF, and were divided into four grades (0, < 1%; 1, 1-10%; 2, 11-30%; 3, > 30%) [Figure 3], in which these cut-offs were determined by the previous studies [14,15]. Level of tissue heterogeneity was taken into considerations. Heterogeneity Score (HeS) was defined as the difference between HS and LS (HeS = HS-LS). The medical charts were reviewed and clinical records including follow up data were collected. Associations between clinico-pathological variables and grading of Ki-67 scoring were analyzed using Chi-square test for categorical variables and Wilcoxon test for continuous variables. Survival probability was estimated using the Kaplan–Meier method and non-parametric group comparison was performed using the log-rank test. Uni- and multivariate analyses were performed using Cox proportional-hazards regression models for age, gender, stage, and HS. Statistical analyses were performed using the JMP 10.0.2 statistical software (SAS institute, Inc., North Carolina, NC). For all tests, *P* -value < 0.05 was considered as significant.

## Results

### Patient demographics

Patient demographics are summarized in Additional file 1. The patients with lung cancer consisted of 68 men and 32 women, with ages ranging from 35 to 88 years (mean 66.3 years) [Additional file 1]. Among the 100 patients with lung cancer, 44 patients died of lung cancer (range of survival time: 6–156 months; mean 44.6 months), 34 were alive (range of follow-up time: 1–62 months, mean 21.6 months), 2 died of other cause, and 20 were lost from follow-up. As for the smoking history, 44 patients were former smokers, with 20 current smokers, 27 never smokers, and 9 cases with unknown smoking history. Pathological stage was available in 91 patients including 43 stage IA, 22 stage IB, 2 stage IIA, 9 stage IIB, 13 stage IIIA, 1 stage IIIB, and 1 stage IV. Among 98 patients, 5 cases were treated with neoadjuvant therapy and 31 cases were treated with adjuvant therapy. The information about the surgical procedures was not available. The histologic classification was as follows: 61 adenocarcinoma, 30 squamous cell carcinoma, 3 small cell carcinoma, 4 large cell carcinoma, and 2 adenosquamous cell carcinoma. From histopathological report, pleural invasion (68 pl0, 12 pl1, 1 pl2, 4 pl3, and 12 data not available), pulmonary metastasis (64 pm0, 23 pm1, and 13 data not available), lymphatic permeation (50 negative, 39 positive, and 11 data not available), and vascular invasion (61 negative, 29 positive, and 10 data not available) were identified.

### Correlation between clinico-pathological variables and Ki-67 scoring

In advance of scoring, 3 small cell carcinomas were excluded from the candidate for evaluation. The HS in Ki-67 staining was 17 score 3, 27 score 2, 28 score 1, 21 score 0, and 4 reels were worn [Table 1]. Several variables including pathological T factor, pathological stage, and vascular invasion had significant differences with HS of Ki-67 scoring (*P* = 0.0051, 0.0384, 0.0265, respectively). And pathological T factor, pathological N factor, pathological stage, pleural invasion, and pulmonary metastasis had significant differences with HeS (P < 0.0001, 0.0008, 0.0041, 0.0010, 0.0420, respectively) [Additional file 1]. LS and AS had no association with any of the variables (data not shown). 78 cases had clinical data including follow-up status. The cases analyzed with all of the data were 74 cases.

**Table 1:** Proportion of score in Ki-67 immunohistochemical staining

	**HS**				**HeS**			
	**0**	**1**	**2**	**3**	**0**	**1**	**2**	**3**
NSCLC (n = 93)	21	28	27	17	30	36	24	3
ADC (n = 58)	17	20	16	5	23	23	13	0
SqCC (n = 27)	3	5	7	12	6	9	9	3

NSCLC, non-small cell lung cancer; ADC, adenocarcinoma; SqCC, squamous cell carcinoma; HS, highest score; HeS, heterogeneity score.

### Correlation of Ki-67 score with overall survival

**Figure 4:**
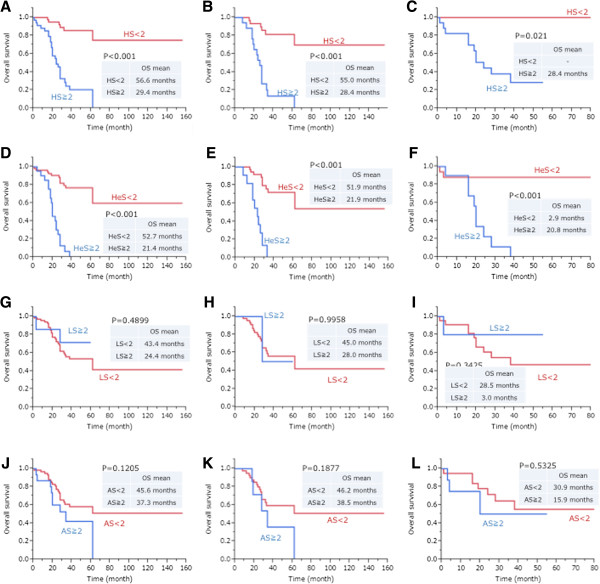
**Kaplan-Meier survival curves for non-small cell lung cancer (NSCLC), adenocarcinoma (ADC), and squamous cell carcinoma (SqCC).** Highest score (HS) 0–1 vs. HS 2–3 in NSCLC **(A)**, in ADC **(B)**, and in SqCC **(C)** show significant prognostic differences between the two groups. Heterogeneity score (HeS) 0–1 vs. HeS 2–3 in NSCLC **(D)**, in ADC **(E)**, and in SqCC **(F)** also show significant prognostic differences between the two groups. Lowest score (LS) 0–1 vs. LS 2–3 in NSCLC **(G)**, in ADC **(H)**, and in SqCC **(I)** and Average score (AS) 0–1 vs. AS 2–3 **(J)** in NSCLC, **(K)** in ADC, **(L)** in SqCC do not show prognostic differences between the two groups.

HS, HeS, LS, and AS were respectively divided into two category which were score 0 or 1 and score 2 or 3. Using by Kaplan-Meier method, overall survival (OS) of patients with HS score 2 or 3 was evidently shorter than patients with a score lower than score 2 with significance (OS mean: HS ≧ 2, 29.4 months; HS < 2, 56.6 months; *P* < 0.001) [Figure 4A]. In addition, the OS of patients with HeS score 2 or 3 was also shorter than patients with a score lower 2 with significance (OS mean: HeS ≧ 2, 21.4 months; HeS < 2, 52.7 months; *P* < 0.001) [Figure 4D], whereas LS or AS did not show any significance in prognostic value [Figure 4G-L]. The results were similar when HS analysis was applied to ADC and SqCC separately (*P* < 0.001 and 0.021, respectively) [Figure 4B,C], and HeS analysis was applied to ADC and SqCC separately (both *P* < 0.001) [Figure 4E,F]. Univariate analyses fitting Cox proportional hazards model adjusted for stage showed significance with OS in NSCLC, ADC, and SCC (*P* < 0.0001, < 0.0001, 0.0055, respectively), and similarly, there was a significant differences between HS and OS in NSCLC, ADC, and SqCC (*P* < 0.0001, < 0.0001, 0.0002, respectively) [Table 2]. In multivariate analyses, stage was significantly associated with OS in NSCLC and ADC (*P* = 0.0002, 0.0024, respectively), and HS had significantly correlation with OS in NSCLC, ADC, and SqCC (*P* = 0.0001, 0.0050, 0.0011, respectively). HS was considered to be an independent prognostic factor of OS in NSCLC, ADC, and SqCC.

**Table 2:** Uni- and multivariate analyses using Cox proportional hazards model

		**Univariate**		**Multivariate**	
		**HR**	***P*-value**	**HR**	***P*-value**
NSCLC (n = 74)	Age	1.021	0.2997	1.014	0.5763
	Gender	1.671	0.1864	1.431	0.3777
	Stage	1.750	*< 0.0001*	1.432	*0.0002*
	HS	8.702	*< 0.0001*	5.497	*0.0001*
ADC (n = 47)	Age	1.033	0.2118	1.004	0.9044
	Gender	1.489	0.3701	1.990	0.1633
	Stage	15.614	*< 0.0001*	1.705	*0.0024*
	HS	7.025	*< 0.0001*	4.424	*0.0050*
SqCC (n = 27)	Age	0.999	0.9942	1.05	0.3197
	Gender	1934	0.2834	1.048e + 10	0.2611
	Stage	12.295	*0.0055*	1.188	0.3096
	HS	1.41e + 9	*0.0002*	1.44e + 10	*0.0011*

NSCLC, non-small cell lung cancer; ADC, adenocarcinoma; SqCC, squamous cell carcinoma; HS, highest score. Significant *P*-values are shown in Italics. *P*-values from log-rank test.

On collecting this cohort, we formed it anonymized. Thus whole slide specimen corresponding the core of Spiral Array was not available and we could not review and reclassified ADC into histological subtypes in new classification of lung adenocarcinoma proposed by International Association for the Study of Lung Cancer (IASLC). In SqCC, we could not evaluate the correlation of Ki-67 expression with the differentiation of SqCC. There is no significance between histopathological subtype, according to WHO classification in 2004, and HS or HeS [Additional file 2].

## Discussion

The correlation of Ki-67 labeling index with the prognosis of neoplasm is reported in many organs [3-7] including lung cancer [14,16]. However, several studies revealed that Ki-67 often fails to be an independent prognostic factor in multivariate analyses [7,16]. And some reports in lung cancer showed limited or negative association to prognosis [17,18]. Our study evidently showed that the highest score (HS) of Ki-67 in NSCLC, ADC and SqCC had correlation with OS by uni- and multivariate analyses. Discordance between previous reports and our results may be due to the evaluation method of Ki-67 and tissue heterogeneity. The meaningful evaluations of biomarkers should be different by tumor types and biomarker. For example, in breast cancer, overall average score is more meaningful [19], in contrast, in neuroendocrine tumors, scoring of highest positive density area shows more benefits [20]. Our data that HS, not Average Score or Lowest Score, only showed significant prognostic difference [Figure 4]. Needless to say, HS is the one that has the strongest confounding nature with tissue heterogeneity. Our data was obtained by new technique named Spiral Array, which covers the morphological variations included in one entire axis of the donor paraffin block [Figure 2]. Morphological consistency between the whole paraffin section and Spiral Array was reported as reasonably higher than conventional tissue microarray [11]. Our data strongly indicates that inadequate scorings for Ki-67 easily lose prognostic value for the tumor, which does have reproducible and strong value by the different scoring methods. Based on our results, it is suggested that the Ki-67 scoring in hotspots, similar to the system proposed in the neuroendocrine tumors may contribute greatly in lung cancer, and overall average Ki-67 scoring like the breast cancer system may not be appropriate. Moreover, in order to take into account tissue heterogeneity, a larger sample might be necessary.

Our study also suggests that levels of tissue heterogeneity (HeS) have correlation with OS (by univariate analysis). Needless to say, our assessment for tissue heterogeneity does not completely cover tissue heterogeneity in the tumor. Potts et al. proposed the more precise method to evaluate tissue heterogeneity by evaluating both cell-level and tumor-level heterogeneity [21]. Compared to the study in which they evaluated the heterogeneity of the protein constantly expressed in the certain cellular location, the marker like Ki-67 by its nature do not possess much importance for the cell-level heterogeneity. Although our HeS is still primitive, this surely shows a certain angle of tissue heterogeneity for the marker like Ki-67. Importantly, we do not think that HeS of Ki-67 can be useful for routine clinic although HeS showed stronger prognostic value than HS but think that HS is more applicable than HeS. However, the fact that higher tissue heterogeneity indicated the poor prognostic impact is interesting. We think that may indicate the presence of higher genetic variables inside the one tumor, which may cause resistance to chemotherapy and/or molecular targeted medicine. Effective and easy applicable way of analyzing tissue heterogeneity along with its reproducible clinical impacts needs to be investigated in the future.

Recently, some molecular markers, including the excision repair cross-complementation group 1 (ERCC1), ribonucleotide reductase M1 (RRM1), epidermal growth factor receptor (EGFR), and ROS1, were revealed as a predictive marker for survival benefit and could also predict the effect of medical treatment [17,18,22]. However, immunohistochemistry (IHC) of these markers are controversial and have a limited use in larger institutions. On the other hand, IHC of Ki-67 was distributed widely, applied to various organs, and established for technique and evaluation of IHC. In this point, IHC of Ki-67 is more common and useful in routine work. Therefore, we considered that evaluation of Ki-67 expression is still important and the results of our study are significant.

## Conclusion

Ki-67 is a strong prognostic marker for non-small cell lung carcinoma when highest staining ratio or degree of heterogeneity is considered. Consideration of tumor heterogeneity is important for the establishment of tissue-based biomarkers.

## Competing interests

Dr. Fukuoka is a representative of a venture company, Pathology Institute Corporation, founded inside Toyama University, and holds stock in the company. Mr. Hori is a director of the same company and also holds stock in the company.

Dr. Fukuoka and Mr. Hori received research funding of $235000 per annum from Sakura Finetek Japan Co, Ltd, from November 2009 to October 2012.

This research was partly funded by the organization of Japan Science and Technology Agency and Japanese Ministry of Health, Labor and Welfare.

No other authors have potential conflicts of interest related to the present work.

## Authors’ contributions

KT participated in the design of the study and performed the statistical analysis, and made draft of the manuscript. TT and SN participated in the collection of clinical information. TH and SY conceived of the study, and participated in its design and coordination and helped to draft the manuscript. TH participated in the selection of cases with Spiral Arrays. JF designed the study, obtained the research funding, carried out the scoring of immunohistochemistry with Ki-67, and wrote the manuscript. All authors read and approved the final manuscript.

## Pre-publication history

The pre-publication history for this paper can be accessed here:

http://www.biomedcentral.com/1472-6890/14/23/prepub

## Supplementary Material

Additional file 1 Characteristics of all 100 lung cancer patients used in this study and correlation with grades of Ki-67 score. Click here for file

Additional file 2 Proportion of score in Ki-67 immunohistochemical staining with 2004 WHO histological classification of Lung Cancer. Click here for file
